# 

**Published:** 1783

**Authors:** 


### PROMOTED.

Lately, John Hunter, Esq. F. R. S. to be one of the foreign associates of the Royal Medical Society at Paris.—Dr. Samuel Stephenson, physician at Berwick, to be a licentiate of the Royal College of Physicians at Edinburgh.

1783. *June* 25. Dr. John Caulet to be a candidate, and Dr. Robert Freer to be a licentiate of the Royal College of Physicians, London.

*July* 1. Mr. Isaac Titford, hospital mate, to be be surgeon to the 2d battalion of the 60th regiment of foot.—Robert Darling Willis, of Caius College, Cambridge, M. A. to be M. B.—9. Messieurs Solomon Berkhead, John Morris, John Watson, and Thomas Waring to be bachelors of physic, and Hugh Shielde, M. D. Edin. to be Doctor of Physic in the university of Philadelphia.—Mr. John Sheldon, surgeon, to be professor of anatomy in the Royal Academy in the room of the late Dr. Hunter.—24. Dr. William Woodville, to be physician to the Middlesex Dispensary in the room of Dr. Watkinson, who resigned on being elected physician to St. Thomas's Hospital.

*August* 4. Dr. John Ellison, of Wakefield in Yorkshire, to be fellow of the Royal College of Physicians at Edinburgh.——9. Mr. Thomas Merrick, late surgeon of the 96th, to be surgeon to the 2d regiment of foot.—20. Mr. Richard Keenlyside to be surgeon to the Newcastle Infirmary in the room of his uncle, Mr. William Keenlyside, deceased.

*Sept.* 1. Dr. Charles Hopson to be physician to the Finsbury Dispensary in London in the room of Dr. Parson, who has resigned.—19. Dr. Gilbert Blane, to be physician to St. Thomas's Hospital, in the room of Dr. Watkinson,

 deceased.

deceased.—Dr. James Carmichael Smyth, F. R. S. to be physician extraordinary to the King.

---

### D I E D.

1782, *March* 13. At Edinburgh, in his 23d year, of a fever, Mr. Jacob Pattison, of Witham in Essex, a student of physic of uncommon genius and assiduity. A monument has been erected to his memory by the companions of his studies, his fellow members in three literary societies; viz. the Medical, the Speculative, and the Physical, of each of which he was president at the time of his death.

1783, *June*. At Hanover, Augustus Hermann Brande, senior, Esq. apothecary to her Majesty.

*July* 23. At Ripon, in Yorkshire, William Richardson, M. D.—31. At Newcastle upon Tyne, Mr. William Keenlyside, senior surgeon to the Infirmary.

*August* 2. In London, aged 60 years, James Buckham, M. D. late physician at Wooller in Northumberland, and author of an Inaugural Dissertation "de Inflammatione ventriculi," printed at Edinburgh in 1758.—3. At Guildford, ford, aged 31 years, James Price, M. D. F. R. S. author of an account of ſome experiments on mercury, ſilver, and gold announced in our 3d vol. p. 328. In 1781 he exchanged his original name of Higginbotham for that of Price, in conformity to the will of a relation, who bequeathed him a handſome fortune.—18. At Worceſter, of the jail fever, James Johnſtone, M. D. author of an Inaugural Diſſertation *de Angina maligna*, printed at Edinburgh in 1773, and publiſhed in Engliſh in 1779, with many judicious additions, under the title of "A "treatiſe on the Malignant Angina, or putrid "or ulcerous ſore throat; with ſome remarks "on the Angina Trachealis."—28. In Crutched Friars, London, aged 40 years, John Watkinſon, M. D. lately elected phyſician to St. Thomas's Hoſpital (ſee p. 211.) He was a native of the county of Eſſex, and ſtudied phyſic firſt at Edinburgh and afterwards at Leyden, at the latter of which places he graduated, He was the author of a pamphlet entitled "An Exami-"nation of a Charge brought againſt Inocula-"tion by de Haen, Raſt, Dimſdale, and other "writers," publiſhed in 1777 in 8vo.—The ſame day, at Northallerton in Yorkſhire, Mr. Wilſon Becket, ſurgeon and apothecary.

Sept. 8.

*Sept.* 3. Mr. Holford, formerly an apothecary at Chelſea.—5. At the houſe of his friend, Dr. Burney, in London, aged 58 years, Mr. William Bewley, ſurgeon and apothecary at Great Maſſingham in Norfolk. He was the author of ſome very ingenious papers addreſſed to Dr. Prieſtley, and publiſhed by him in his obſervations on different kinds of air, and in his experiments and obſervations relating to various branches of natural philoſophy.—21. In Great George-ſtreet, Hanover-ſquare, of a pulmonary conſumption, aged 26 years, George Edward Hawkins, Eſq. (ſon of Pennel Hawkins, Eſq.) ſurgeon to the King's houſehold and to St. George's Hoſpital.—At Stockton, aged 87, Mr. Robert Smith, formerly a ſurgeon of the navy.

---

### SECTION IV.

### QUARTERLY CATALOGUE.

1. OBſervations on the ſuperior efficacy of the Red Peruvian Bark in the cure of agues and other fevers, interſperſed with occaſional remarks on the treatment of other diſeaſes by the ſame remedy. Third edition, with con-

ſiderable additions, and an appendix, containing a more particular account of its natural hiſtory. By *William Saunders*, M. D. F. A. S. member of the Royal College of Phyſicians in London, and phyſician to Guy's Hoſpital. 8vo. *Johnſon*, London, 1783. 188 pages.

Our readers will find an account of the firſt edition of this work in our 3d vol. p. 248. The preſent edition contains much additional information on the ſubject by Dr. Fothergill, the late Dr. Keir, Dr. Maddocks, Dr. Simmons, Mr. Aikin, Mr. Rigby, and Mr. Shireff.—The reader will find an extract from Dr. Simmons's letter, relative to the natural hiſtory of this Bark, under the head of Medical News, in page 305.

2. Letters on the Medical ſervice of the Royal Navy; with occaſional remarks, including new obſervations on the general practice of phyſic, and the beſt means of preſerving the health of his Majeſty's ſeamen. To which is added a recapitulation of the correſpondence between the author and Dr. Hawes, on a ſubject of the firſt importance to the welfare of ſociety. By *W. Renwick*, ſurgeon of the Royal Navy. 8vo. *Newbery*, London, 1783. 2s.

3. Practical obſervations on the human teeth. By *R. Wooffendale*, ſurgeon-dentiſt, Liverpool. 8vo. *Johnſon*, London, 1783. 158 pages.

4. Experiments and obſervations in Electricity. By *Thomas Milner*, M. D. 8vo. *Cadell*, London, 1783. 111 pages with 2 copper-plates. 2s.

6. Flora Diætetica, or the hiſtory of Eſculent plants, both domeſtic and foreign, in which they are accurately deſcribed, and reduced to their Linnæan Generic and ſpecific names; with their Engliſh names annexed, and ranged under eleven general heads; viz. 1. roots; 2. ſhoots, ſtalks, &c. 3. leaves; 4. flowers; 5. berries; 6. ſtone-fruit; 7. apples; 8. legumens; 9. grain; 10. nuts; 11. funguſes; and a particular account of the manner of uſing them; their native place of growth; their ſeveral varieties and phyſical properties: together with whatever is otherwiſe curious, or very remarkable in each ſpecies. The whole ſo methodized as to form a ſhort introduction to the ſcience of Botany. By *Charles Bryant* of Norwich. 8vo. *White*, London, 1783. 375 pages.

7. A treatiſe on the Venereal diſeaſe. By *James Dunbar Innes*, A. M. ſurgeon in London, and member of the Royal Medical Society of Edinburgh. 8vo. *Cumberledge*, London, 1783. 100 pages. 2s.

7. Obſervations ſur le traitement de la Gonorrhée, traduites de l'Anglois de M. *Samuel Foart Simmons*, Docteur en Medecine, membre du College Royal des Medecins, et de la Société Royale de Londres; Aſſocié etranger de la Société Royale de Medecine de Paris, &c. 8vo. Paris, 1783. 67 pages.

This accurate tranſlation of Dr. Simmons's work on the gonorrhœa is written by M. Andry, doctor regent of the faculty of phyſic at Paris. For an account of the original work the reader is referred to our 2d vol. p. 23.

8. Analyſe du fer, par M. *Tob. Bergman*, Chevalier de l'Ordre Royal de Vaſa; traduite du Latin en François, avec des notes et un appendice, et ſuivie de quatres memoires ſur la metallurgie. *i. e.* Analyſis of iron, by Sir *Thorbern Bergman*, knight of the royal order of Waſa; tranſlated from the Latin into French, with notes and an appendix, with the addition of four eſſays on metallurgy. By *M. Grignon*, knight of the order of St. Michael, and correſpondent of the R. A. of Sciences. 8vo. Paris, 1783. 302 pages.

Not contented with giving a faithful tranſlation of this elaborate treatiſe, and many uſeful notes, the Chevalier Grignon, in an appendix, exhibits at one view the results of the numerous experiments contained in the work.

9. Vollstoendiges verzeichniss aller gewœsche Deutschlands, &c. *i. e.* A complete catalogue of all the plants of Germany, vol. I. 8vo. Leipsic, 1782.

This is an anonymous publication, but it is printed at the expence of the Society of Natural Historians at Berlin. This circumstance will be sufficient to recommend it to the notice of botanical readers. The plants are arranged according to the Linnæan system. References are given to all the German *Flora*'s hitherto published, and to the works in which the best engravings of the different plants are to be met with.

10. Verzeichniss und beschreibung der Tyroler insekten, *i. e.* A catalogue and description of the insects of Tyrol. By *J. Nepomucene von Laicharting*. Part I. containing the genus *Scarabæus*. 8vo. Zurich, 1782. 248 pages.

11. Principios, &c. *i. e.* Principles of botany drawn from the best writers, and translated into Spanish. By Don *Michael Barnades*, M. D. formerly physician to his majesty and first professor of botany. 8vo. Madrid, 1783.

12. Es-

12. Especifico, &c. *i. e.* Specific newly discovered in the kingdom of Guatimala, for the radical cure of cancer and other more frequent complaints. By Don *Joseph Flores*, M. D. To which is added, a copy of a letter written at Mexico the 25th of May last, by a person worthy of credit, concerning the good effects of this specific in that city. 8vo. Madrid, 1782.

13. Bibliotheca Chirurgica, in quâ res ad Chirurgiam pertinentes ordine alphabetico, ipsi vero scriptores quotquot ad annum usque 1779 innotuerunt, ad singulas materias ordine chronologico adhibentur, adjecto ad libri calcem auctorum codice, studio et operâ *Stephani Hieronymi de Vigiliis von Creutzenfeld*, Phil. & Med. Doct. Fac. Med. Vindob. Memb. 4to tom. II. Vindobonæ, 1781.

This work was undertaken at the desire of Baron Stoerck, to whom it is dedicated. It is preferable to Haller's *Bibliotheca Chirurgica* both in the arrangement of the materials, and in its being brought down to a later period, viz. to 1779 instead of 1775.

14. Versuche aus der theoretischen arzneykunste, *i. e.* Essay on the theory of physic. By *John Ulrich Gottlieb Schæffer*, M. D. counsellor of ſtate and body phyſician to the prince of Oeting. 8vo. Nuremberg, 1782. 125 pages.

15. Praktiſche paſtoral arzneykunſt, *i. e.* A paſtoral practice of phyſic for the uſe of the clergy. By *John Nepomucene Anthony Leuthner*, M. D. counſellor of ſtate and body phyſician to the elector palatine, aſſeſſor of the college of phyſicians at Munich, &c. 8vo. Nuremberg, 1782. 358 pages.

16. Inſtitutiones Neurologicæ, ſive de nervis corporis humani tractatio; præmiſſa eſt oratio de proprietatibus nervorum generalioribus, publice habita in regia Academiâ Suecana. Editio altera recentiorum obſervationibus aucta et priori emendata. Auctore *Rollando Martin*, M. D. anatom. et chirurg. et theatro anatom. urbis metropol. prof. olim et reg. colleg. med. aſſeſſor. 8vo. Stockholm, 1782. p. 113.

17. Inſtitutiones Neurologicæ, ſive de nervis. Addita eſt ſciagraphia nervorum tabulis XI. comprehenſa. Lectio ſecunda anatomica. Auctore *Rollando Martin*, M. D. &c. 8vo. Stockholm, 1782. p. 250.

18. Recherches ſur la petite verole, ſa marche, ſes nuances, et les meilleurs moyens de la traiter; avec des obſervations ſur l'epidemie qui a regné dans l'Anfreville et les environs; ſur la nature nature des gas inflammables et detonnans, et les meilleurs moyens de prevenir leurs effets pernicieux ou d'y remedier; et fur la dyfenterie epidemique qui a regné l'année 1779, dans la ville de Caen et aux environs. *i. e.* Inquiries concerning the fmall pox, its progrefs, its diftinctions, and the beft methods of treating it; with remarks on the epidemic that has prevailed at Anfreville and its neighbourhood; on the nature of inflammable and detonating airs, and the beft means of preventing or obviating their pernicious effects; and on the epidemic dyfentery that prevailed in the year 1779, in the city of Caen, and its environs. By *H. F. A. de Rouffel*, M. D. profeffor of phyfic in the univerfity of Caen, member of the academy of Belles Lettres of that city, and of the college of phyficians at Lyons. 8vo. Caen, 1783. 200 pages.

19. Entwurf einer Verzeichnung veteranifcher bucher. *i. e.* Plan of a catalogue of books relative to the veterinary art. By *John Charles Gottlieb Henze.* 8vo. Gottingen, 1782.

20. Verfuche uber die Platina. *i. e.* Effays on Platina. 8vo. Manheim, 1782. 324 pages.

Thefe effays are the production of comte de Sickingen, minifter from the elector palatine to the court of France. The author has communicated nicated them in French to the academy of sciences at Paris, and they will appear in the *Memoires des savans etrangers.* In the mean time he has permitted this German translation of them to be published. They contain a great number of experiments on a large scale, which afford many new lights on the subject of Platina.

21. Dissertatio Medica Inauguralis de Phthisi Pulmonali, auctore *Gulielmo Corp*, Anglo. 8vo. Edin. 1782.

22. Dissertatio Medica Inauguralis de Plethora, auctore *Joanne Radulpho Fenwick*, Anglo. 8vo. Edin. 1782.

23. Dissertatio Medica Inauguralis de Erysipelate, auctore *Gulielmo Gourlay*, Scoto. 8vo. Edin. 1782. p. 40.

24. Dissertatio Medica Inauguralis de Morbis Mammarum, auctore *Edvardo Hart*, Hiberno. 8vo. Edin. 1782.

25. Dissertatio Medica Inauguralis de Melæna, auctore *Harper Hall*, ex Insula Barbada. 8vo. Edin. 1782.

26. Dissertatio Medica Inauguralis de Typho Graviore Petechiali, auctore *Georgio Paton*, Scoto. 8vo. Edin. 1782. p. 38.

27. Dissertatio Medica Inauguralis de Menorrhagia, rhagia, auctore *Andrew Sayers*, Hiberno. 8vo. Edin. 1782.

28. Dissertatio Inauguralis de aere dephlogisticato, auctore *Jonathan Stokes*, Anglo. 8vo. Edin. 1782. p. 40.

29. Dissertatio Medica Inauguralis de animi affectibus, auctore *Daniel Bryan*, Hiberno. 8vo. Edin. 1782.

30. Dissertatio Medica Inauguralis de cynanche typho, auctore *Georgio Daniel*, Anglo. 8vo. Edin. 1782.

31. Dissertatio Inauguralis de aeris affectibus, auctore *Samuel de Butts*, Hiberno. 8vo. Edin. 1782.

32. Dissertatio Medica Inauguralis de febre puerperarum, auctore *Thoma Evory*, Hiberno. 8vo. Edin. 1782.

33. Dissertatio Medica Inauguralis de Pneumonia, auctore *Jacobo Forsythe*, Hiberno. 8vo. Edin. 1782.

34. Dissertatio Inauguralis de Catarrho, auctore *Henrico Garde*, Hiberno. 8vo. Edin. 1782.

35. Dissertatio Medica Inauguralis de Syncope, auctore *Jacobo Hare*, Scoto. 8vo. Edin. 1782.

36.

36. Dissertatio Inauguralis de Mente. Auctore *Philippo Holland*, Anglo. 8vo. Edin. 1782. p. 35.

37. Dissertatio Inauguralis de febrium mutatione. Auctore *Jacobo Hutchinson*, Britanno. 8vo. Edin. 1782.

38. Dissertatio Medica Inauguralis de Militum Salute tuenda. Auctore *Andrea Marshall*, Scoto. 8vo. Edin. 1782.

39. Dissertatio Medica Inauguralis de rheumatismo. Auctore *Ricardo Vaughan*, Anglo. 8vo. Edin. 1782.

40. Recherches sur la nature et le traitement de la fievre puerperale, ou inflammation d'entrailles des femmes en couches. *i. e.* Inquiries concerning the nature and treatment of the puerperal fever, or abdominal inflammation of lying-in women. By M. *de Laroche*, physician to the Duke of Orleans, member of the College of Physicians of Geneva, and of the Royal Medical Society of Edinburgh. 12mo. Paris, 1783. 332 pages.

41. Instructions concernant les femmes enceintes, celles qui sont accouchées, et la maniere d'elever les petits enfans, avec les moyens d'eviter l'abus, et les prejugés funestes qui les font perir trop ordinairement. *i. e.* Instructions concerning cerning pregnant and lying-in women, and the manner of educating little children, with the means of avoiding the fatal miſtakes and prejudices which too commonly prove fatal to them. By M. *Saucerotte*, member of ſeveral academies. 12mo. Straſburg, 1783. 99 pages.

42. Verſuch eines neuen Lehrgobæudes der praktiſchen Geburthyhülfe. *i. e.* An Eſſay towards a new ſyſtem of the practice of midwifery. By *John Philip Hagen*, profeſſor of midwifery at Berlin, &c. Part I. 211 pages. Part II. 276 pages. 8vo. Berlin, 1782.

In this work the author gives the reſult of his own practice. He has delivered 350 women, of whom 26 died; 111 of theſe women were in their firſt pregnancy, and brought forth 117 children (three of them having had twins) of whom only 64 were born alive. The total number of children produced by the 350 women amounted to 364, of whom 134 were ſtill-born. Thirty-nine women were delivered with Levret's forceps, and twenty-eight with the crotchet. This practice, at firſt view, may to the Engliſh practitioner appear very unſucceſsful; but it muſt be obſerved, that, upon the Continent, accoucheurs are ſeldom called in except in difficult caſes.—The inſtruments employed by the author

 conſiſt confift of Levret's forceps, a forceps for the feet, of his own invention, and of which an engraving is given, a crotchet and blunt hook. A plate is likewife added of a chair and a bed proper for women in labour.

43. Guide ou manuel dans le traitement des maladies les plus graves & les plus frequentes. *i. e.* A Guide or manual for the treatment of difeafes the moft dangerous and the moft frequent. 8vo. Paris, 1782. 383 pages.

This anonymous publication is faid to be the production of the late M. Sylva, doctor-regent of the Faculty of Phyfic at Páris. It has confiderable merit, fo far as relates to the defcriptions of difeafes; but that part of it which treats of the methods of cure feems to be lefs deferving of praife.

44. Eloge Hiftorique de Jean Bafeilhac, dit Frere Cofme, religieux Feuillant et chirurgien lithotomifte, avec des details fur les inftrumens qu'il a inventés, ou perfectionnés, pour la taille dans le haut et le bas appareil et autres operations chirurgicales. *i. e.* Hiftorical Eulogy of John Bafeilhac, commonly called Friar Come, a monk of the order of Feuillants, and furgeon-lithotomift, with an account of the inftruments he invented or perfected for lithotomy, either by the the high or the low operation, or for other chirurgical operations. By *M. Cambon*, furgeon to her late R. H. the princefs Charlotte of Lorraine. 8vo. Paris, 1781. 31 pages.

45. Handbuch der Naturgefcheichte. *i. e.* A Text-book of Natural Hiftory. By *John Frederick Blumenbach*, M. D. 8vo. Gottingen, 1780.

46. *Jo. Chr. Gottl. Ackermann* de Dyfenteriæ antiquitatibus liber bipartitus. 8vo. Leipfic.

47. Pieces intereffantes fur la Medecine et la Phyfique, favoir, 1. Le regime Pythagoricien, pour vivre en parfait fanté jufqu'à une extreme vieilleffe; 2. Difcours fur l'hiftoire naturelle; 3. Defcription du corps humain; 4. Differtation fur les forces de l'imagination; 5. Les differens fyftemes fur la generation; 6. Mefure et calcul des douleurs et des plaifirs; 7. Difcours fur la fympathie, traduit de Cocchi et autres celebres medecins. *i. e.* Interefting pieces relative to Phyfic and Natural Philofophy, viz. 1. On the Pythagorician regimen, to live in perfect health even to extreme old age; 2. Difcourfe on natural hiftory; 3. Defcription of the human body; 4. Differtation on the powers of the imagination; 5. The different fyftems of generation; 6. Scale and calculation of pains and pleafures;

 7. Dif-

7. Difcourfe on fympathy, tranflated from Cocchi and other celebrated phyficians. 12mo. Paris, 1782. 371 pages.

48. Leçons elementaires d'Hiftoire Naturelle et de Chemie, dans lefquelles on s'eft propofé, 1. de donner un enfemble methodique des connoiffances chymiques acquifes jufqu'à ce jour; 2. d'offrir un tableau comparé de la doctrine de Stahl et de celle de quelques modernes, pour fervir de refumé à un cours complet fur ces deux fciences. *i. e.* Elementary Lectures on Natural Hiftory and Chemiftry, the intention of which is, 1. to give a methodical view of the knowledge acquired in chemiftry to the prefent time; and, 2. to offer a comparative table of the doctrine of Stahl and of that of certain modern chemifts; the whole being defigned as a text-book to a complete courfe on thefe two fciences. By *M. de Fourcroy*, doctor regent of the faculty of phyfic, and member of the Royal Medical Society. 8vo. Paris, 1782. Vol. I. 584 pages. Vol. II. 848 pages.

The defign of this work is to convey only a few general ideas of Natural Hiftory, fo that the greater part is allotted to Chemiftry. The author's view is to connect thefe two fciences, and and to point out how much a knowledge of chemiſtry is requiſite for natural hiſtory, and *vice verſa.* The whole work is divided into ſeventy lectures, and in all of them he carefully diſtinguiſhes facts from theory.

49. Diſſertation ſur l'importance des evacuans dans la cure des plaies recentes, ou gravés; ſuivie d'obſervations raiſonnées ſur la complication du vice venerien et ſcorbutique. *i. e.* A Diſſertation on the importance of evacuations in the cure of wounds either recent or dangerous; to which are added, caſes (with remarks) of the complication of the venereal virus with ſcurvy. By *M. Lombard*, correſponding member of the Royal Academy of ſurgery, and ſurgeon-major to the military hoſpital at Straſburgh. 8vo. Straſburgh, 1782. 160 pages.

The author quotes the authority of Hippocrates, Galen, and Boerhaave in oppoſition to Guy de Chauliac, Pigray, Ambroſe Paré, and ſome other writers who have maintained, that emetics and purgatives retard ſuppuration and the healing of wounds. He points out the circumſtances in which they may be employed with advantage, and relates ſeveral inſtances of their good good effects, particularly in wounds of the head.

He then offers fome remarks on the complication of the venereal with the fcorbutic virus; on the preference which cauftics claim over the knife in the opening of venereal buboes, and on the abufe of relaxing and greafy applications to thefe fores.

50. Hiftoire des Maladies epidemiques qui ont regné dans la province du Dauphiné depuis l'année 1775. *i. e.* Hiftory of the epidemical difeafes that have prevailed in the province of Dauphiny fince the year 1775. By *M. Nicolas*, Med. & Philof. Doct. Confulting phyfician to the king for the treatment of epidemics, &c. 8vo. Grenoble, 1780. 110 pages.

51. Suite de l'Aitiologie de la Salivation, ou explication des inconveniens attachés au mercure adminiftré en friction et en fumigation; avec des obfervations fur les dangers de l'ufage du fublimé corrofif, et fur ceux de toutes les preparations du mercure données fous forme feche. *i. e.* Sequel to the Ætiology of Salivation, or an explanation of the inconveniencies annexed to the adminiftration of mercury in friction or fumigation; with remarks on the dangers arifing from the ufe of corrofive fublimate; and on thofe thoſe of all the preparations of mercury given in a dry form. By *John Staniſlaus Mittié*, doctor regent of the faculty of phyſic at Paris, and member of the royal academy of ſciences at Nancy, and phyſician to the late King Staniſlaus. 8vo. Paris, 1781. 157 pages.

This is intended as a ſupplement to a work formerly publiſhed by the author on ſalivation.

52. Collection Academique, compoſée des memoires, actes, ou journaux des plus celebres academies et ſociétés litteraires de l'Europe, concernant l'hiſtoire naturelle, la botanique, la phyſique, la chemie, la medecine, l'anatomie, la mechanique, &c. *i. e.* Academical Collection, compiled from the memoirs, tranſactions, or journals of the moſt celebrated academies and literary ſocieties of Europe, concerning natural hiſtory, botany, natural philoſophy, chemiſtry, phyſic, anatomy, mechanics, &c. Vol. 6th, containing a continuation of the Hiſtory and Memoirs of the Royal Academy of Sciences at Paris. 4to. Paris, 1781. 596 pages, with plates.

53. Traité de l'Anthrax, ou de la Puſtule maligne. *i. e.* A treatiſe on the Anthrax or malignant Puſtule. By M. *Chambon*, doctor-regent of the Faculty of Phyſic, and member of of the Royal Medical Society at Paris. 12mo. Neufchatel., 1782, 230 pages.

54. Differtation fur le charbon malin de la Bourgogne, ou la puftule maligne. *i. e.* Differtation on the malignant carbuncle of Burgundy, or malignant puftule, being the work that obtained the premium given by the Academy of Dijon. By M. *Thomaffin*, furgeon-major of the firft regiment of light horfe. 8vo. Bafil, 1782. 2d edition. 72 pages.

44. Recherches fur differens points de phyfiologie, de pathologie et de Therapeutique, pour fervir de bafe à un cours de pathologie. *i. e.* Inquiries on different points of phyfiology, pathology, and Therapeutics, intended as a bafis to a courfe of pathology. By M. *Fabre*, Regius Profeffor in the College of Surgery. 8vo. Paris, 1783.

55. Kleine Medinifch Chirurgifch Abhandlungen. *i. e.* Opufcula of Phyfic and Surgery tranflated from different languages. Vol. I. 8vo. Leipfic, 1782. 249 pages.

